# A Low Peripheral Blood CD4/CD8 Ratio Is Associated with Pulmonary Emphysema in HIV

**DOI:** 10.1371/journal.pone.0170857

**Published:** 2017-01-25

**Authors:** Matthew Triplette, Engi F. Attia, Kathleen M. Akgün, Guy W. Soo Hoo, Matthew S. Freiberg, Adeel A. Butt, Cherry Wongtrakool, Matthew Bidwell Goetz, Sheldon T. Brown, Christopher J. Graber, Laurence Huang, Kristina Crothers

**Affiliations:** 1 Department of Medicine, University of Washington, Seattle, Washington, United States of America; 2 Department of Medicine, Veterans Affairs Connecticut Healthcare System, New Haven, Connecticut, United States of America; 3 Department of Internal Medicine, Yale University School of Medicine, New Haven, Connecticut, United States of America; 4 Department of Medicine, Veterans Affairs Greater Los Angeles Healthcare System, Los Angeles, California, United States of America; 5 Department of Medicine, David Geffen School of Medicine at UCLA, Los Angeles, California, United States of America; 6 Department of Medicine, Vanderbilt University, Nashville, Tennessee, United States of America; 7 Department of Medicine, Weill Cornell Medical College, New York, New York, United States of America; 8 Hamad Healthcare Quality Institute and Medical Corporation, Doha, Qatar; 9 Department of Medicine, Atlanta Veterans Affairs Medical Center, Atlanta, Georgia, United States of America; 10 Department of Medicine, Emory University School of Medicine, Atlanta, Georgia, United States of America; 11 Department of Medicine, James J. Peters Veterans Affairs Medical Center, New York, New York, United States of America; 12 Department of Medicine, Icahn School of Medicine at Mt. Sinai, New York, New York, United States of America; 13 Department of Medicine, University of California San Francisco, San Francisco, California, United States of America; University of Kansas Medical Center, UNITED STATES

## Abstract

**Objectives:**

The prevalence of emphysema is higher among HIV-infected (HIV+) individuals compared to HIV-uninfected persons. While greater tobacco use contributes, HIV-related effects on immunity likely confer additional risk. Low peripheral blood CD4+ to CD8+ T-lymphocyte (CD4/CD8) ratio may reflect chronic inflammation in HIV and may be a marker of chronic lung disease in this population. Therefore, we sought to determine whether the CD4/CD8 ratio was associated with chronic obstructive pulmonary disease (COPD), particularly the emphysema subtype, in a cohort of HIV+ subjects.

**Methods:**

We performed a cross-sectional analysis of 190 HIV+ subjects enrolled in the Examinations of HIV Associated Lung Emphysema (EXHALE) study. Subjects underwent baseline laboratory assessments, pulmonary function testing and chest computed tomography (CT) analyzed for emphysema severity and distribution. We determined the association between CD4/CD8 ratio and emphysema, and the association between CD4/CD8 ratio and pulmonary function markers of COPD.

**Results:**

Mild or greater emphysema (>10% lung involvement) was present in 31% of subjects. Low CD4/CD8 ratio was associated with >10% emphysema in multivariable models, adjusting for risk factors including smoking, current and nadir CD4 count and HIV RNA level. Those with CD4/CD8 ratio <0.4 had 6.3 (1.1–39) times the odds of >10% emphysema compared to those with a ratio >1.0 in fully adjusted models. A low CD4/CD8 ratio was also associated with reduced diffusion capacity (DLCO).

**Conclusions:**

A low CD4/CD8 ratio was associated with emphysema and low DLCO in HIV+ subjects, independent of other risk factors and clinical markers of HIV. The CD4/CD8 ratio may be a useful, clinically available, marker for risk of emphysema in HIV+ subjects in the antiretroviral therapy (ART) era.

## Introduction

The prevalence of emphysema and other chronic lung diseases is higher in HIV-infected (HIV+) compared to HIV-uninfected (HIV-) persons. [1–5] Although greater tobacco use accounts for much of this increase in risk, HIV infection is an independent risk factor for chronic obstructive pulmonary disease (COPD), particularly the emphysema subtype. The link underlying the association of HIV and emphysema remains incompletely understood, but may be related to immune dysfunction, severity of immunocompromise induced by HIV infection, and HIV-related immune activation. [1,3]

A low peripheral blood ratio of CD4+ to CD8+ T-lymphocytes (CD4/CD8) has recently been described as a marker of ongoing immune activation in HIV+ patients despite treatment with antiretroviral therapy (ART). [6,7] Readily available clinically, the CD4/CD8 ratio has been associated with markers of inflammation and senescence, and with increased incidence of and mortality from chronic diseases in patients with HIV. [6–10] A low CD4/CD8 ratio in the lung mucosa has been associated with both the development and the severity of COPD in HIV, but there is little evidence regarding the significance of the peripheral blood CD4/CD8 ratio in lung disease in HIV+ persons. We have previously found that elevated plasma levels of soluble CD14 (sCD14), a marker of monocyte activation, were associated with emphysema in HIV+ but not HIV- subjects, supporting a potential role of immune activation in the development of emphysema among those with underlying HIV. [4]

The peripheral blood CD4/CD8 ratio may serve as a marker of chronic immune activation, and may be clinically useful in identifying HIV+ individuals at risk for chronic lung disease related to increased inflammation, particularly emphysema. Our primary aim in this study was therefore to determine the association between CD4/CD8 ratio and radiographic emphysema. Our secondary aims were to determine the association between CD4/CD8 ratio and other markers of COPD and pulmonary dysfunction in HIV including airflow obstruction, forced expiratory volume in one second (FEV1), and diffusion capacity (DLCO). We hypothesized that a low CD4/CD8 ratio would be associated with emphysema and with pulmonary function markers of COPD, independent of other markers including the current and nadir CD4 cell count.

## Materials and Methods

### Study Design and Population

We performed a cross-sectional analysis of subjects enrolled in the Examinations of HIV Associated Lung Emphysema (EXHALE) study, a pulmonary substudy of the Veterans Aging Cohort Study (VACS). [11] The VACS cohort included approximately 3500 HIV+ and 3500 age-, race-, sex-, and site-matched HIV- Veterans at eight Veterans Affairs (VA) Medical Centers (VAMC) in the United States. EXHALE recruited VACS subjects at four of these VAMC (Atlanta, Bronx, Houston and Los Angeles) to participate in a pulmonary substudy, enrolling subjects from 2009–2012. HIV+ and HIV- outpatients enrolled in VACS were approached for enrollment into EXHALE while undergoing clinical care at the VAMC, and were block matched by smoking status (current vs. non-current smokers). Subjects were excluded if they had chronic lung diseases other than COPD or asthma, or had acute respiratory illnesses. 385 HIV+ and HIV- subjects were consented for enrollment into EXHALE and 366 completed any baseline testing. For this study, we restricted our analyses to the HIV+ subjects (n = 196) in EXHALE who had baseline measures of both CD4 and CD8 cell counts (n = 190).

The Institutional Review Boards of the following institutions approved this study: VA Connecticut Healthcare System, Michael E. DeBakey VA Medical Center, VA Greater Los Angeles Healthcare System, Atlanta VA Medical Center, James J. Peters Veterans Affairs Medical Center and the University of Washington. All subjects provided written informed consent.

### Primary Definition of Emphysema

Of the 190 HIV+ subjects, 164 underwent chest CT with research interpretation. For the emphysema outcome, we restricted the analytic cohort to these 164 subjects, as data imputation for emphysema severity is not appropriate. CT scans were performed at a participant’s baseline visit using a multidetector CT scanner at one of the four sites. The CT protocol was standardized across the different scanners, which included a Phillips Brilliance 40 (Atlanta), Siemens Sensation 64 (Bronx, Los Angeles), and Siemens Sensation Cardiac 16-detector (Houston). The CT data were acquired at a moderate radiation exposure (100 mAs) without radiopaque contrast with subjects holding his/her breath at end-inspiration in the supine position. CT images were reconstructed using both moderate and high-spatial frequency kernels to create thin section images (< 1.0 mm). CT scanner compliance evaluations were performed prior to study initiation and annually using two custom-made phantoms consisting of the three carbon foam sections designed to mimic lung tissue density (Bitca Lung Phantom, Bitca Ltd, Alberta, Canada). The mean and standard deviation of the pixel values in predefined regions of the phantoms were computed to compare scanner performance. No evidence of drift in Hounsfield units out of an acceptable range was observed during the annual scanner compliance evaluations.

A single thoracic radiologist interpreted the scans, blinded to clinical history and HIV status, and assigned a score for emphysema severity based on visual inspection. Scores ranged from 0 to 5 based on semi-quantitative assessment of emphysema: 0 (no emphysema), 1 (1–10% emphysema), 2 (>10–25% emphysema), 3 (>25–50% emphysema), 4 (>50–75% emphysema), and 5 (>75% emphysema). For this study, we dichotomized the variable as >10% emphysema (mild or greater emphysema) or ≤10% (trace to no emphysema) for the main analyses, similar to prior studies. [4,12] We selected this dichotomized score as it represents the median score in our cohort, and there were few patients with the highest 2 categories of emphysema severity (n = 13 for subjects with emphysema >50%). We examined the trend of CD4/CD8 ratio across the 5 emphysema severity categories using Cuzick’s non-parametric test for trend [13] and differences in distribution and pattern of emphysema by CD4/CD8 ratio group.

### Definition of Pulmonary Function Variables

Pre- and post-bronchodilator spirometry and DLCO (corrected for hemoglobin) were obtained for each subject at baseline in the clinical pulmonary function testing laboratories affiliated with the participating VAMC. All spirometry and DLCO measurements were reviewed to ensure that they met American Thoracic Society (ATS) standards for acceptability and reproducibility. We calculated percent-predicted values using standardized reference equations from the National Health and Nutrition Examination Survey (NHANES) III, using the Hankinson equation for spirometry and the Neas and Schwartz equation to calculate percent-predicted DLCO including adjustment for hemoglobin. [14,15] We also made additional adjustments by racial and ethnic categorization per the current literature for racial groups (i.e. Native Americans, Asians or Native Hawaiians/Pacific Islanders) not included in NHANES III (n = 2 participants). [16–18]

### CD4/CD8 Ratio and Other Data Collection

The CD4 and CD8 cell counts were obtained from routine clinical measurements in the VA electronic health record (EHR) closest to enrollment. The median time from enrollment date to CD4 and CD8 measurement was 29 days (IQR 8–50). CD4/CD8 ratio was stratified into three groups (<0.4, 0.4–1, >1.0; low, middle, normal) for the primary analysis. This stratification was determined *a priori* based on previously published literature on the CD4/CD8 ratio: a normal ratio is >1.0; a ratio of <1.0 has been associated with an immune profile reflective of increased mortality in an HIV- population; and a ratio <0.4 may be most useful to predict serious non-AIDS events in HIV+ patients. [8,19] Demographic information for each subject was obtained from VA administrative databases. Smoking was defined based on a standard questionnaire at enrollment. [5] Smoking status was defined as never-smoker, current-smoker, or former smoker (at least 100 lifetime cigarettes and quit >12 months prior). For each subject, pack-years of cigarette use was calculated based on average number of cigarettes smoked per day and years smoked. Other laboratory values, including HIV RNA level (viral load), were also collected at baseline from the VA EHR. Nadir CD4 counts reflected the lowest available VA laboratory value prior to enrollment. Plasma biomarkers, including sCD14, were measured at enrollment (Laboratory for Clinical Biochemistry Research, University of Vermont). Antiretroviral therapy (ART) use was obtained from VA pharmacy data. Other comorbid illnesses were based on International Classification of Diseases 9 codes (available at www.vacohort.org).

### Statistical Analysis

We initially stratified baseline characteristics by categories of CD4/CD8 ratio as well as dichotomized emphysema severity (>10% or ≤10% involvement). We used Wilcoxon rank sum or Kruskal-Wallis testing, where appropriate, for continuous variables presenting medians with interquartile ranges or χ^2^ testing for categorical variables. We then created multivariable logistic regression models to determine the independent association between CD4/CD8 ratio and >10% emphysema. Models were adjusted for variables that we determined *a priori* may confound the relationship between CD4/CD8 and emphysema or chronic lung disease, namely: demographics, chronic diseases (chronic heart disease, hypertension, diabetes and anemia), cigarette pack-years and history of injection or inhalational drug use. To determine if the CD4/CD8 ratio was associated with emphysema independent of other markers of HIV disease severity (i.e., CD4 count and HIV viral load), we incorporated these values into a mutually adjusted model. Although CD4 count is part of the CD4/CD8 ratio, these variables are not perfectly collinear when incorporated into the same model as separate predictors with CD4 count as a continuous variable. In a third model, we determined whether CD4/CD8 ratio association was independent of the subject’s nadir CD4 count as a marker of prior immunosuppression and potential for immunologic recovery, as well as sCD14, another marker of immune activation (in this case, monocyte activation) previously associated with emphysema in a subset of this cohort. [4]

In secondary analyses, we determined the association between CD4/CD8 ratio and other markers of chronic lung disease (percent-predicted DLCO, percent-predicted FEV1 and airflow obstruction). We defined airflow obstruction as a post-bronchodilator FEV1/forced vital capacity (FVC) ratio <0.7, per Global Initiative for Chronic Obstructive Lung Disease (GOLD) guidelines. [20] We created multivariable linear regression models (for DLCO and FEV1) and logistic regression models (airflow obstruction) for each outcome of interest, adjusted for covariates as above.

We conducted sensitivity analyses to further define the relationship between CD4/CD8 ratio and emphysema. First, we created robust models of CD4/CD8 ratio, incorporating linear, quadratic and cubic terms and we also compared participants in the lowest ratio category to the remaining participants. Second, we created a model including only subjects with an undetectable viral load to determine the association with emphysema in this group with viremia suppression.

All results were considered significant at a two-sided p-value <0.05. Analyses were performed using Stata version 14.0 (College Station, TX, USA).

## Results

The majority of the subjects were male (98%). The median age at baseline was 55 (IQR 49–59) years old. Most subjects were black (72%); 13% of subjects were white and 12% identified as Hispanic. Most were either current (63%) or former (21%) smokers. 71% of subjects were on ART at study entry; 66% had an undetectable viral load and 86% had a CD4 cell count ≥200 cells/μL.

### Bivariate Analysis by CD4/CD8 Ratio and Emphysema Status

We first stratified our cohort by CD4/CD8 ratio categories (Table 1). Of the 190 subjects, 71 (37%) had a ratio <0.4, 84 (44%) had a ratio of 0.4–1.0, and 35 (19%) had a ratio >1.0. There were no significant associations between demographic characteristics, chronic diseases, smoking behavior, drug use and pneumonia history with CD4/CD8 ratio. Low CD4 and high CD8 counts, as parts of the CD4/CD8 ratio, were highly associated with a low ratio as was a detectable viral load and a nadir CD4<200 cells/μL.

**Table 1:** HIV+ subject characteristics by CD4/CD8 ratio groups (n = 190).

Variable	CD4/CD8<0.4 (n = 71)	CD4/CD8 0.4–1.0 (n = 84)	CD4/CD8>1.0 (n = 35)	p-value*
Demographics and comorbidities				
Male (%)	100	99	94	0.08
Age	53 (49–58)	56 (49–58.5)	56 (51–63)	0.33
Race				0.37
Black (%)	77	71	60	
White (%)	13	12	14	
Hispanic (%)	7.0	14	17	
BMI	26 (24–30)	26 (23–30)	26 (24–29)	0.92
Chronic heart disease (%)	18	14	17	0.79
Diabetes (%)	14	19	34	0.05
Hypertension (%)	48	58	57	0.40
Anemia (%)	15	15	20	0.81
History of TB (%)	11	2.4	5.7	0.08
History of PCP (%)	2.8	2.4	0	0.62
History of bacterial pneumonia (%)	24	15	8.6	0.12
History of TB, PCP or bacterial pneumonia (%)	31	19	14	0.09
Smoking and drug use				
Smoking status				0.30
Never (%)	14	18	11	
Former (%)	14	25	26	
Current (%)	72	57	63	
Pack-years	22 (8.0–42)	15 (2.8–36)	18 (11.4–37)	0.40
History of IVDU (%)	32	33	34	0.97
History of inhalational drug use (%)	87	88	91	0.80
HIV-related				
CD4 (cells/μL)	280 (156–407)	493 (378–636)	687 (486–874)	<0.001
CD4<200 cells/μL (%)	32	3.6	2.9	<0.001
Nadir CD4 <200 cells/μL (%)	75	56	32	<0.001
CD8 (cells/μL)	1237 (907–1582)	832 (653–1086)	474 (376–701)	<0.001
Viral load >50 copies/mL (%)	52	30	8.8	<0.001
Soluble CD14 (ng/mL)	1568 (1411–1953)	1669 (1386–2087)	1655 (1356–2069)	0.81
On ART (%)	75	70	66	0.62
Pulmonary function and emphysema				
>10% emphysema (%)	43	25	21	0.03
FEV1/FVC, pre-bronchodilator	75 (66–81)	75 (67–79)	76 (70–79)	0.79
FEV1/FVC, post-bronchodilator	75 (70–82)	78 (71–83)	78 (75–82)	0.55
Airflow obstruction (%)	25	23	9	0.20
FEV1% pred, pre-bronchodilator	85 (75–99)	87 (74–104)	93 (83–102)	0.24
FEV1% pred, post-bronchodilator	90 (82–104)	92 (79–110)	94 (86–103)	0.45
FVC % pred, pre-bronchodilator	91 (83–103)	92 (82–108)	97 (87–105)	0.45
FVC % pred, post-bronchodilator	96 (86–104)	96 (84–108)	96 (89–105)	0.96
DLCO % pred	51 (41–58)	53 (44–67)	59 (49–71)	0.008

Continuous variables presented as medians (interquartile range) and categorical variables presented as %.

BMI = body mass index; IVDU = intravenous drug use; TB = tuberculosis; PCP = *Pneumocystis* pneumonia; FEV1 = forced expiratory volume in 1 second; FVC = forced vital capacity, % pred = percent-predicted; DLCO = diffusion capacity of carbon monoxide; ART = antiretroviral therapy

*P-value is for χ^2^ or Kruskal Wallis testing between 3 categories of CD4/CD8 ratio

**Fig 1:**
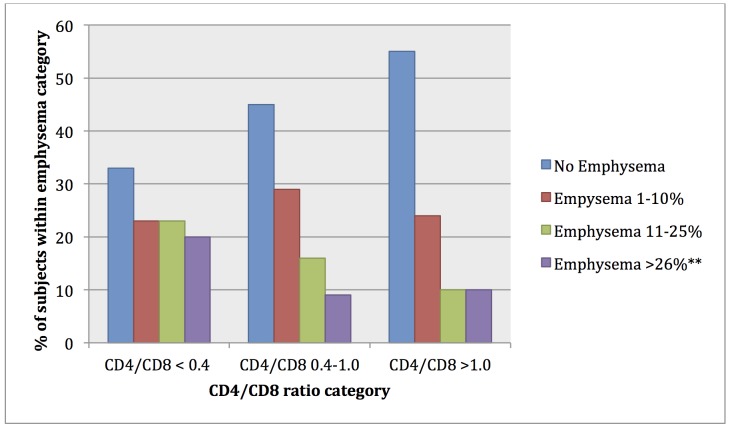
Severity of emphysema by CD4/CD8 ratio group (p = 0.02 for trend)*. * Cuzick’s test of trend **Emphysema categories of 26–50%, 51–75% and >75% collapsed for this graph given low numbers of subjects in higher emphysema categories.

Subjects with lower CD4/CD8 ratio were more likely to have >10% emphysema: 43% of those with CD4/CD8 ratio <0.4 had emphysema, compared to 25% of those with CD4/CD8 ratio 0.4–1.0 and 21% of those with CD4/CD8 >1.0 (p = 0.03 for χ^2^ test). The anatomic distribution of emphysema was similar between groups of CD4/CD8 ratio as was pattern of emphysema (i.e. centrilobular, panlobular and with or without paraseptal emphysema), with the majority of subjects with >10% emphysema having diffuse centrilobular disease involving all lobes. Using Cuzick’s test of trend, there was also a significant trend towards higher severity of emphysema (0–5 scores) in lower CD4/CD8 ratio groups (p = 0.02) (Fig 1). Lower CD4/CD8 ratio was also significantly associated with lower DLCO. Airflow obstruction and spirometry values (FEV1 and FVC) were not significantly different between the groups.

We then stratified our cohort by presence of emphysema, comparing those with >10% (n = 51) to those with ≤10% emphysema (n = 113) (Table 2). As expected, those with >10% emphysema were older and had lower BMI, greater smoking histories, more inhalation drug use and were more likely to report prior pulmonary infection. Those with emphysema >10% were also more likely to have airflow obstruction, a lower FEV1/FVC ratio, lower DLCO, and lower pre- and post-bronchodilator FEV1. In this cohort, emphysema was not associated with baseline CD4 count or viral load, but was significantly associated with a nadir CD4 count <200 cells/μL; 76% of those with emphysema versus 53% of those without had a prior CD4 count <200 cells/μL. The median CD4/CD8 ratio for those with >10% emphysema was 0.38 (IQR 0.25–0.59), compared to 0.54 (IQR 0.33–0.88) (p = 0.03) for those with ≤10% emphysema.

**Table 2:** Subject characteristics by semi-quantitative emphysema>10% (n = 164).

Variable	Emphysema>10% (n = 51)	Emphysema≤10% (n = 113)	p-value*
Demographics and comorbidities			
Male (%)	98	98	0.93
Age	57 (52–61)	54 (47–58)	0.03
Race			0.07
Black (%)	76	71	
White (%)	20	12	
Hispanic (%)	4	12	
BMI	24 (21–26)	27 (24–31)	<0.001
Chronic heart disease (%)	16	18	0.75
Diabetes (%)	14	25	0.11
Hypertension (%)	49	56	0.42
Anemia (%)	14	17	0.62
History of TB (%)	12	5.3	0.14
History of PCP (%)	3.9	0.90	0.18
History of bacterial pneumonia (%)	27	12	0.01
History of TB, PCP or bacterial pneumonia (%)	15	29	0.001
Smoking and drug use			
Smoking status			0.04
Never (%)	6.0	19	
Former (%)	18	23	
Current (%)	76	58	
Pack-years	34 (13–48)	15 (2.7–32)	<0.001
History of IVDU (%)	34	30	0.59
History of inhalational drug use (%)	96	85	0.04
HIV-related			
CD4/CD8 ratio (linear)	0.38 (0.25–0.59)	0.54 (0.33–0.88)	0.03
CD4/CD8 ratio			0.03
<0.4 (%)	51	30	
0.4–1 (%)	37	50	
>1.0 (%)	12	20	
CD4 (cells/μL)	383 (241–591)	455 (324–636)	0.09
CD4<200 cells/μL (%)	20	11	0.12
Nadir CD4<200 cells/μL (%)	76	53	0.008
CD8 (cells/μL)	907 (592–1362)	937 (663–1237)	0.81
Viral load>50 copies/mL (%)	39	33	0.48
Soluble CD14 (ng/mL)	1674 (1487–2164)	1626 (1364–1953)	0.13
On ART (%)	76	73	0.68
Pulmonary Function and Emphysema			
FEV1/FVC, pre-bronchodilator	68 (62–76)	78 (72–81)	<0.001
FEV1/FVC, post-bronchodilator	71 (65–77)	80 (76–84)	<0.001
Airflow obstruction (%)	44	7.4	<0.001
FEV1% pred, pre-bronchodilator	83 (70–93)	93 (79–105)	0.003
FEV1% pred, post-bronchodilator	87 (76–101)	97 (84–109)	0.005
FVC % pred, pre-bronchodilator	96 (82–107)	93 (84–105)	0.63
FVC % pred, post-bronchodilator	97 (86–111)	96 (86–104)	0.40
DLCO % pred	45 (39–57)	56 (48–70)	<0.001

Continuous variables presented as medians (interquartile range) and categorical variables presented as %.

BMI = body mass index; IVDU = intravenous drug use; TB = tuberculosis; PCP = *Pneumocystis* pneumonia; FEV1 = forced expiratory volume in 1 second; % pred = percent-predicted; DLCO = diffusion capacity of carbon monoxide; ART = antiretroviral therapy

*P-value is for χ^2^ or Wilcoxon rank sum testing

### Multivariable Analysis of Associations between CD4/CD8 Ratio and Emphysema

In multivariable logistic regression analyses adjusting for demographics, smoking, BMI, chronic diseases and drug use, those with CD4/CD8 ratio <0.4 had 6.0 (95% CI 1.6–22) times the odds of >10% emphysema compared to those with CD4/CD8 ratio >1.0 (Table 3). In a second multivariable model, now with additional adjustment for CD4 count and HIV viral load as continuous variables, those with CD4/CD8 ratio <0.4 remained at higher risk for emphysema (OR 7.4, 95% CI 1.5–35) compared to those with CD4/CD8 ratio >1.0. The addition of markers previously associated with emphysema in our cohort, nadir CD4 cell count and sCD14, to the model did not attenuate the association between CD4/CD8 ratio and emphysema (OR 6.3, 95% CI 1.1–39 for CD4/CD8<0.4 compared to CD4/CD8>1.0). Neither nadir CD4 count nor sCD14 maintained a significant association with emphysema. HIV+ subjects with CD4/CD8 ratio between 0.4–1.0 did not have significantly different odds of emphysema compared to those with a higher ratio in any of these models.

**Table 3:** Associations between CD4/CD8 ratio category (relative to ratio>1.0) with emphysema and airflow obstruction in logistic regression models.

Outcome	CD4/CD8<0.4	CD4/CD8 0.4–1.0
**>10% emphysema**^a^ Unadjusted model Adjusted model^b^ Adjusted model plus CD4 and HIV viral load^c^ Adjusted model plus CD4, HIV viral load, nadir CD4 and sCD14^d^	2.9 (1.0, 8.2)* 6.0 (1.6, 22)* 7.4 (1.5, 35)* 6.3 (1.1, 39)*	1.3 (0.46, 3.7) 1.3 (0.37, 4.5) 1.4 (0.38, 5.1) 1.2 (0.27, 5.5)
**Airflow obstruction**^a^ Unadjusted model Adjusted model^b^ Adjusted model plus CD4 and HIV viral load^c^	3.2 (0.85, 123) 7.1(1.4, 35)* 3.5 (0.61, 20)	2.81 (0.77, 10) 4.6 (1.0, 22)* 3.5 (0.72, 17)

^a^Odds ratios (95% CI) presented for CD4/CD8 ratios relative to ratio >1.0

^b^Model adjusted for age, sex, race/ethnicity, BMI, pack-years of tobacco use, chronic diseases (chronic heart disease, anemia, diabetes, hypertension) and inhalational and intravenous drug use

^c^Adjusted model with additional inclusion of CD4 cell count and viral load

^d^Adjusted model with additional inclusion of CD4 cell count, viral load, nadir CD4 and soluble CD14

*P<0.05

### Association between CD4/CD8 Ratio and DLCO, FEV1 and Airflow Obstruction

In multivariable linear regression models, the percent-predicted DLCO was also associated with CD4/CD8 ratio (Table 4). The estimated DLCO percent-predicted was 8.7% (95% CI 3.1%-14%) lower among those with CD4/CD8 ratio <0.4 compared to those with CD4/CD8 ratio >1.0. In models also adjusted for current and nadir CD4 cell count, viral load and sCD14, the estimated DLCO percent-predicted remained significantly lower when comparing a CD4/CD8 ratio <0.4 to a CD4/CD8 ratio >1.0. In both of these models, the percent-predicted DLCO was non-significantly lower when comparing groups with CD4/CD8 ratio of 0.4–1.0 to those with a ratio >1.0. There was a general trend towards a lower FEV1 with lower CD4/CD8 ratio group, but this was not significant in any models. In unadjusted models, CD4/CD8 ratio <0.4 was not associated with airflow obstruction, but the relationship was significant in adjusted models: compared to those with a CD4/CD8 ratio >1.0, those with a ratio <0.4 had an increased risk of airflow obstruction (OR 7.1, 95% CI 1.4–35), and those with CD4/CD8 ratio 0.4–1.0 also had a significantly increased risk of airflow obstruction (OR 4.6, 95% CI 1.0–22). However, these associations were attenuated in models adjusting for current CD4 cell count and HIV viral load, and were no longer statistically significant.

**Table 4:** Associations between CD4/CD8 ratio categories (relative to ratio>1.0) with DLCO and FEV1 in linear regression models.

Outcome	CD4/CD8<0.4	CD4/CD8 0.4–1.0
**DLCO %-predicted**^a^ Unadjusted model Adjusted model^b^ Adjusted model plus CD4 and HIV viral load^c^ Adjusted model plus CD4, HIV viral load, nadir CD4 and sCD14^d^	-11% (-17%, -4.5%)* -8.7% (-14%, -3.1%)* -8.7% (-14%, -2.9%)* -9.0% (-17%, -1.4%)*	-5.5% (-12%, 0.7%) -5.0% (-10%, 0.36%) -5.0% (-10%, 0.47%) -5.7% (-12, 0.67)
**FEV1%-predicted**^a^ Unadjusted model Adjusted model^b^ Adjusted model plus CD4 and HIV viral load^c^	-5.1% (-13%, 3.1%) -7.4% (-16%, 1.0%) -3.4% (-14%, 6.7%)	-3.1% (-11%, 4.9%) -5.1% (-13%, 2.9%) -3.7% (-12.%, 4.7%)

^a^Estimate of difference in %-predicted values (95% CI) for CD4/CD8 ratios relative to ratio >1.0

^b^Model adjusted for age, sex, race/ethnicity, BMI, pack-years of tobacco use, chronic diseases (chronic heart disease, anemia, diabetes, hypertension) and inhalational and intravenous drug use

^c^Adjusted model with additional inclusion of CD4 cell count and viral load

^d^Adjusted model with additional inclusion of CD4 cell count, viral load, nadir CD4 and soluble CD14

*P<0.05

DLCO = diffusion capacity of carbon monoxide, FEV1 = forced expiratory volume in 1 second

### Sensitivity Analyses

In sensitivity analyses, we modeled the CD4/CD8 measure in different ways to assess the robustness of our results. Quadratic and cubic terms for the ratio were evaluated but did not add to model fit. When CD4/CD8 ratio was included as a continuous measure, this model performed similarly to the models above. In adjusted models, a decrease in ratio of 1.0 (i.e. a decrease in CD4/CD8 ratio from 1.5 to 0.5) was associated with 4.1 (95% CI 1.5–12) times the odds of emphysema and remained statistically significant when current CD4 and HIV viral load were added to the model (OR 4.6, 95% CI 1.2–17). We also compared emphysema risk in subjects in the lowest CD4/CD8 ratio category (<0.4) to the remaining patients. 43% of patients in this lowest category had emphysema compared to 24% of subjects with a ratio ≥0.4 (p = 0.01).

We also sought to determine whether results were consistent in subjects with HIV viral load suppression (n = 105). In adjusted analyses among those with viral load suppression, those with CD4/CD8 <0.4 (n = 36) were estimated to have 20 times the odds (95% CI 2.5–153) of emphysema. This association persisted after adjustment for CD4 cell count and viral load (OR 30, 95% CI 2.7–331).

## Discussion

In this study, we sought to determine whether a decreased CD4/CD8 ratio was associated with emphysema and other pulmonary function markers of COPD. In our cohort of 190 HIV+ subjects, we found that a low CD4/CD8 ratio was associated with semi-quantitative radiographic emphysema and this association was independent of potential confounding factors including smoking, age, other major chronic diseases, current and nadir CD4 cell count, and HIV viral load. This association was robust when modeling the CD4/CD8 ratio in different ways, as a continuous measure or in categories. We also found a significant trend towards higher severity of emphysema in those with lower CD4/CD8 ratios (Fig 1). Additionally, we found a statistically significant association between a low CD4/CD8 ratio with low DLCO, but no definitive association between a low CD4/CD8 ratio with airflow obstruction and FEV1. These findings reinforce that CD4/CD8 ratio is associated with emphysema, which is defined radiographically but is also characterized by–and correlated with–a low DLCO. [21,22]

The CD4/CD8 ratio is thought to reflect residual inflammation and immune activation in HIV+ patients, and may be correlated with emphysema in HIV by way of these mechanisms, which may persist despite ART. [23] The CD4/CD8 ratio falls dramatically in early HIV infection, reflecting a decline in CD4 count and a proliferation of HIV-specific CD8+ T-cells. With effective ART, most patients experience a reconstitution of CD4+ T-cells and many have a decline in the CD8+ T-cells with a normalized ratio. However, prior studies demonstrate that a subset of patients has a persistently depressed CD4/CD8 ratio. This can reflect ongoing depression in CD4+ T-cell counts; however, many patients maintain CD8+ T-cell expansion despite a normalization of the CD4+ T- cell count and a suppression of viral load. [23–27] In recent cohort and case-control studies, a low CD4/CD8 ratio has been associated with markers of T-cell activation (HLADR+, CD38+), and early senescence (CD57+, CD28-) as well as poor clinical outcomes including increased incidence, morbidity and mortality from serious non-AIDS-related events. [6–10]

The CD4/CD8 ratio within the lung has been associated with pulmonary-specific inflammation and lung disease in previous studies. A low CD4/CD8 ratio and elevated CD8+ T-cells have been demonstrated in airway and pulmonary parenchymal samples in patients from the general population with COPD. [28,29] Early studies in HIV lung disease also demonstrated elevated alveolar CD8+ lymphocytes in HIV-associated lymphocytic alveolitis and an association with increased viral burden in the lung, but did not explore an association with chronic lung disease. [30–32] However, in a recent study, Popescu and colleagues examined the CD4/CD8 ratio in bronchoalveolar mononuclear cells in a small cohort of HIV+ and HIV-uninfected subjects and found a low CD4/CD8 ratio was associated with COPD severity in those with HIV. [33]

The association between the peripheral blood CD4/CD8 ratio and chronic pulmonary disease, either in the general population or those with HIV, remains unclear. In small studies, a low CD4/CD8 ratio has been found in certain subsets of patients with COPD including non-smokers, those with airflow obstruction but preserved DLCO, those experiencing an exacerbation of COPD and those with decreased expression of alpha-1 antitrypsin. [34–37] Low CD4/CD8 ratio was also linked with a higher burden of respiratory symptoms in a cohort of subjects with COPD. [38] There are few studies that examine the peripheral blood CD4/CD8 ratio and lung disease in HIV+ patients. In their study of airway CD4/CD8 ratio, Popescu and colleagues did not find a statistically significant association between the peripheral blood CD4/CD8 ratio and COPD; however, their cohort of 27 HIV+ subjects was limited by its small sample size. [33]

We were most interested in the association between the CD4/CD8 ratio and emphysema because increasing evidence suggests that emphysema, linked to chronic inflammation in the general population, [39,40] may be particularly associated with residual inflammation in those with HIV. Emphysema is more prevalent in HIV, and has been associated with markers of systemic inflammation and immunosuppression (nadir CD4 cell count <200) in HIV+ patients. Emphysema has also been associated with extrapulmonary inflammatory diseases, such as non-obstructive coronary artery disease, in HIV. [41] Of note, in this study we found a trend towards higher sCD14, in subjects with emphysema but no significant association, which may challenge the role of monocyte-specific inflammation in emphysema among those with HIV. [4]

We demonstrate that the peripheral blood CD4/CD8 ratio, as a marker that may reflect residual immune activation in HIV, is associated with radiographic emphysema in our cohort of HIV+ subjects. The additional significant finding of the independent association of a low CD4/CD8 ratio and DLCO provides further evidence of a link with emphysema. These findings indicate that the peripheral CD4/CD8 ratio mirrors what has been demonstrated in bronchoalveolar lavage studies. The CD4/CD8 does not solely reflect chronic immune activation and inflammation (as it can be low simply as a function of low CD4), but we have demonstrated that in the subset with HIV control and viral load suppression, the ratio remains a significant marker for emphysema. We suggest that these findings may indicate that systemic inflammation and immune activation, reflected by a low CD4/CD8 ratio, may lead to increased parenchymal destruction that is characteristic of emphysema, however, this would require further investigation. Given the cross-sectional nature of the study, we cannot determine whether the low CD4/CD8 ratio is contributing to emphysema pathogenesis or whether emphysema drives a low CD4/CD8, as the CD4/CD8 ratio could be depressed in the setting of local inflammation and tissue destruction from emphysema. Regardless, the CD4/CD8 ratio may be a useful marker of risk of emphysema.

Our study has certain limitations. Our cohort is the largest we are aware of to examine the association between the peripheral blood CD4/CD8 ratio and chronic pulmonary disease, with carefully characterized CT scan and pulmonary function test data; however, our study remained limited by power. In a larger cohort, it may have been possible to demonstrate a more robust relationship between the CD4/CD8 ratio and our secondary outcomes, specifically low FEV1 and airflow obstruction, which were less common in our study compared to emphysema and low DLCO. While our findings were statistically significant, the confidence intervals were wide, with sample size limiting the precision of the estimates of the magnitude of associations. Also, while our ratio groupings were based on published data, there is some discrepancy in the literature regarding the threshold that constitutes a higher risk CD4/CD8 ratio. Our results suggest that the CD4/CD8 is associated with emphysema both as a continuous measure and at a threshold ratio <0.4, but these analyses were not intended to suggest that a ratio of 0.4 is optimal for defining HIV+ patients at highest risk for chronic diseases such as emphysema. Finally, all subjects were Veterans and largely male, limiting generalizability, although our study cohort was racially and geographically diverse.

In conclusion, in our cohort of HIV+ subjects we found an independent association of a low peripheral blood CD4/CD8 ratio with radiographic emphysema and a low DLCO that is likely reflective of pulmonary parenchymal loss in emphysema. The CD4/CD8 ratio has been linked to ongoing inflammation and immune activation in HIV. The association between CD4/CD8 ratio with emphysema in HIV suggests that HIV-specific inflammatory pathways may be involved in the development or progression of emphysema, however, this will require further study. These results also suggest that the CD4/CD8 ratio may be useful as a clinical marker of risk for emphysema in HIV+ patients.

## Supporting Information

S1 File CD4/CD8 Codebook. This is the codebook guide for the dataset.
(XLSX)

S2 File CD4/CD8 Dataset. This is the dataset.
(XLS)
